# Simultaneous EEG-fMRI for Functional Neurological Assessment

**DOI:** 10.3389/fneur.2019.00848

**Published:** 2019-08-13

**Authors:** Giulia Mele, Carlo Cavaliere, Vincenzo Alfano, Mario Orsini, Marco Salvatore, Marco Aiello

**Affiliations:** IRCCS SDN, Naples, Italy

**Keywords:** EEG, fMRI, multimodal image analysis, functional connectivity, EEG spectra

## Abstract

The increasing incidence of neurodegenerative and psychiatric diseases requires increasingly sophisticated tools for their diagnosis and monitoring. Clinical assessment takes advantage of objective parameters extracted by electroencephalogram and magnetic resonance imaging (MRI) among others, to support clinical management of neurological diseases. The complementarity of these two tools can be now emphasized by the possibility of integrating the two technologies in a hybrid solution, allowing simultaneous acquisition of the two signals by the novel EEG-fMRI technology. This review will focus on simultaneous EEG-fMRI technology and related early studies, dealing about issues related to the acquisition and processing of simultaneous signals, and including critical discussion about clinical and technological perspectives.

## Introduction

The incidence of neurodegenerative and psychiatric diseases has increased in the last decades, requiring finer, and advanced tools, ranging from electrophysiology to neuroimaging, for a reliable diagnostic accuracy. Electrophysiology, and specifically the electroencephalogram (EEG), represents a consolidated, and widespread tool supporting the diagnosis of neurological diseases. Unlike imaging techniques, EEG offers an excellent temporal resolution, recording the electric brain activity in the order of milliseconds through electrodes placed on the scalp. Through EEG signal processing techniques, and dedicated experimental setup, quantitative parameters on the spectrum of frequencies, amplitudes, and coherence can be achieved.

Conversely, computed tomography (CT), and mainly MRI, provide a morphological view of brain (1), with an excellent spatial resolution, allowing a multiparametric assessment of the brain tissue properties, both in terms of structural and functional information. In this context, similarly to EEG but at different temporal scales (milliseconds vs. seconds), functional MRI (fMRI) allows for non-invasive investigation of brain functional activation both during resting state and task execution, enriching the panel of parameters achievable by MRI (e.g., structural connectivity revealed by diffusion tensor imaging, metabolites concentrations revealed by magnetic resonance spectroscopy, and perfusion revealed by arterial spin labeling). This complementarity of information is deeply exploited by multimodal acquisition systems that are developed to overcome single modality drawbacks and to improve the compliance of the patients. Both in preclinical and clinical settings (2–6), first multimodal imaging techniques attempted to combine functional information derived by nuclear medicine modalities (positron emission tomography—PET, and single photon emission computed tomography—SPECT) with structural data achieved by CT and MRI, in order to complement diagnostic and prognostic approach to different kind of patients (7). In neurology, simultaneous PET/MRI paved the way for a more comprehensive investigation of brain organization and physiology, allowing to investigate, within a single integrated exam, the cerebral connectivity in terms of structural, functional, and metabolic connectome (8, 9). Recently, to fully investigate healthy and pathological brain function, novel tools have been developed to simultaneously acquire EEG and fMRI signals, integrating the optimal temporal and spatial resolution of both techniques and overcoming the limitations of single modalities.

In this review, simultaneous EEG-fMRI technology, detailing current applications using both resting state and task approaches and discussing future perspectives will be focused.

## EEG

**Figure 1:**
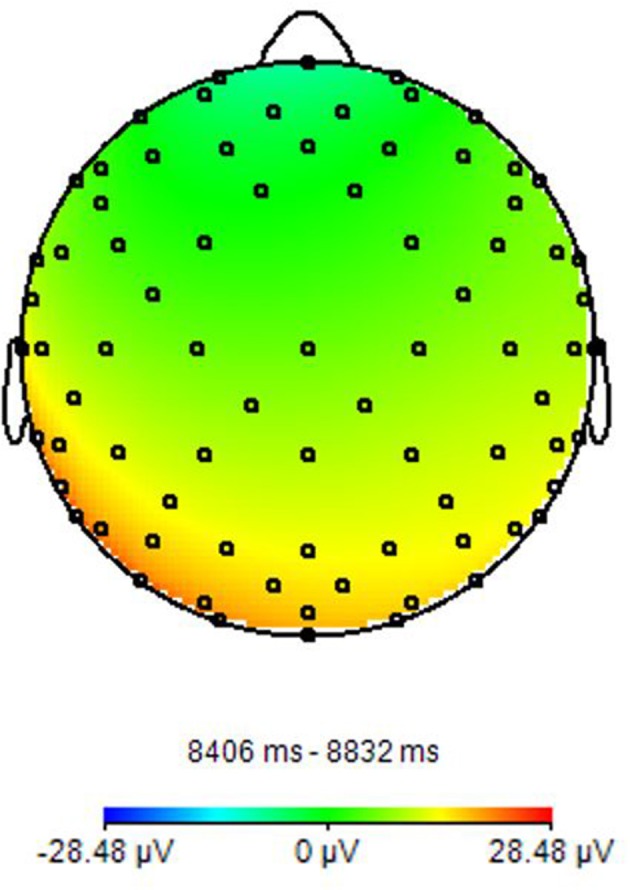
EEG power spectrum. It presents a Topographic representation of Alpha power activity. Image obtained on a 40 years-old healthy volunteer with hybrid EEG-fMRI system and included for illustrative purpose only.

EEG is one of the most used techniques for studying brain electrical activity. The first acquisition of an electroencephalograph was made more than 50 years ago by Berger, who recorded brain electrical activity via a radio equipment. The discovery of EEG and, consequently, of cerebral electrical activity definitely changed the way of approaching to the study of brain structures and functions, and over the time became a fundamental tool in both clinical and research fields (10). Brain electrical activity is derived from the synchronizations of a pool of cortical neurons, in particular of pyramidal cells. These cells present a different electrical charge along the neuron, resulting negative on dendrites and positive in the rest of cell. This difference determines an electric dipole that can be acquired by EEG electrodes, and represented as a series of positive and negative waves. However, the electric field derived by a single pyramidal cell is not enough to obtain a detectable EEG signal. For this reason, the electrodes record a pool of cells arranged parallel to each other, and producing radial and tangential dipoles (11, 12). The EEG is acquired through the positioning of electrodes on the scalp according to the international 10–20 system (13), which takes into account four main reference point: nasion, inion, and the two preauricular points (A1, A2) (14). The electrodes are fixed to the scalp by means of a conductive paste and recorded a lot of brain oscillations including delta rhythm (0.5–4 Hz), theta rhythm (4–8 Hz), alpha rhythm (8–13 Hz), beta rhythm (13–30 Hz), and gamma rhythm (above 30 Hz) (15) (Figure 1) Moreover, during the task execution, it is possible to record evoked potentials that allow to study different neuronal processes (16). The evoked potentials can be divided according to latency. In fact, the potentials that occur within the 100 ms post stimulus are usually due to the nature of the stimulus itself, while the subsequent components reflect the cognitive processes related to the perception of the stimulus (Shravani et al., 2009).Technological innovations have led to the development of high-density EEG systems with a high number of channels/electrodes for quantitative EEG and brain connectivity studies (17). Currently, within a clinical setting (configuration with about 20 electrodes), the EEG is used to characterize numerous diseases including metabolic or drug alterations, sleep disorders, epileptic syndromes, neurodegenerative diseases, traumatic brain injury, and tumor lesions, and the characterization of comatose patients and brain death (18).

## fMRI

The fMRI is one of the main non-invasive techniques that allow to measure brain function. The mechanism that subtends the signal of fMRI is called blood oxygen level dependent (BOLD) effect, that describes the variation in the magnetic status of the red blood cells linked to the hemoglobin oxygenation. Indeed, the form of hemoglobin without oxygen is deoxyhemoglobin, which has paramagnetic property, while oxyhemoglobin has diamagnetic property. In resting conditions, the balance between these two elements concentrations in the vascular brain system, provides a signal indistinguishable from the surrounding parenchyma. When a stimulus was applied, the hemoglobin balance in specific brain areas changes, initially in favor to deoxyhemoglobin concentration and so determining a decrease of signal, and following switching in favor to oxyhemoglobin concentration and a signal increase (19). The detection of these signal changes translates into a series of images, that can be analyzed to show the activations of specific brain areas, following the execution of specific tasks. It is important to understand that BOLD effect is an indirect measure of neuronal activation, depending from neurovascular coupling and so by different interplay, such as alteration in blood flow and volume and complex interactions between the activated neurocircuitry with astrocytic and vascular targets. Briefly, neuronal activation induced by the stimulus determines a neurotransmitter release in the synaptic cleft and its uptake by the astrocytic process, in the so-called tripartite synapse (20, 21). The secondary astrocytic activation triggers intracellular Ca2+fluctuations in astrocyte end-feet that elicit cellular molecular and hemodynamic changes recorded by fMRI through the release of vasoactive peptides (22). This complex cascade of events that subtend neurovascular coupling and BOLD effect is also responsible of the time delay between neuronal activation and BOLD signal fluctuation that distinguish fMRI from direct electrophysiological measures.

In this context, while task-related fMRI has been applied in many studies to investigate specific functions and/or brain areas (23), more recently, resting-state fMRI approach is coming out to analyze spontaneous physiological fluctuations without the need of patient's compliance, pathway's integrity and command following, sometime impossible in several kind of patients (24, 25).

Since from its development, fMRI technique has been applied to characterize brain functional connectivity in several physiological conditions (26, 27) and many diseases, including brain tumors (28), multiple sclerosis (29), Alzheimer's diseases (AD) (30, 31), epilepsy (32), but also psychiatric disorders (33, 34).

## Simultaneous EEG-fMRI

Simultaneous EEG-fMRI acquisition is used to evaluate the correlation between electrical brain activity and hemodynamic mutation. fMRI with high spatial resolution does not provide adequate temporal sampling due to the slow BOLD response (in order of seconds) unlike EEG that instead offers a high temporal resolution (in the order of milliseconds), but with a poor localization of signal sources (35). The integration of these two tools in a hybrid simultaneous acquisition allows to overcome the intrinsic limitations of both the techniques and to increase the plethora of analyses that can be performed, and in turns, of the information that can be achieved (36). Simultaneous acquisition also guarantees an identical registration, as regards the mental state of the subject, the execution of the task and the inference of the recording environment. This does not happen by recording the two methods separately, especially if the recording takes place in different environments and with cognitive unstable patients (37).

As for technological issues, the acquisition of simultaneous EEG/fMRI involves the use of specialized EEG hardware that is safe and compatible with the MR environment and comfortable to the participant. Improper use of the equipment may result in considerable risks. Regarding safety, a potential risk for the subjects comes from electrodes and heating of conducting leads during MR radio frequency transmission, resulting in discomfort or even burns (38). To reduce the risk of subject discomfort or injuries, there are several precautions, for example fMRI sequences should be based on gradient echo-echo planar imaging (GE-EPI); for anatomical reference scans, low specific absorption rate (SAR) sequences should be used, in particular GE-T1-weighted sequences; for all sequences in EEG-fMRI protocol, it should be ascertained that their SAR does not exceed the SAR of the GE-EPI sequence. Otherwise, extensive safety testing with temperature sensors is necessary. Staff performing EEG-MRI studies must have received appropriate training, as injuries due to MR-compatible EEG equipment cannot be ruled out if the equipment is accidentally used out of specifications, especially in the case of body coil transmission (39). The adoption of these guidelines is particularly important in vigilance-reduced subjects (sleeping or sedated subjects) or, generally, in subjects who cannot give notice of any discomfort reliably (children).

Regarding the compliance of the subjects, it is important when using EEG/fMRI to make sure that they have a good understanding of all steps involved, that they are comfortable with all steps, and that there are no accidents that could cause discomfort leading to movement and resulting in failure of the experiment (40). The participants should understand that nothing will be painful even if some steps may be slightly uncomfortable, such as slight abrasion of the scalp during placement of EEG electrodes; this helps eliminate much of the anxiety that the participant might otherwise have, in order to complete the experiment properly and safely.

**Figure 2:**
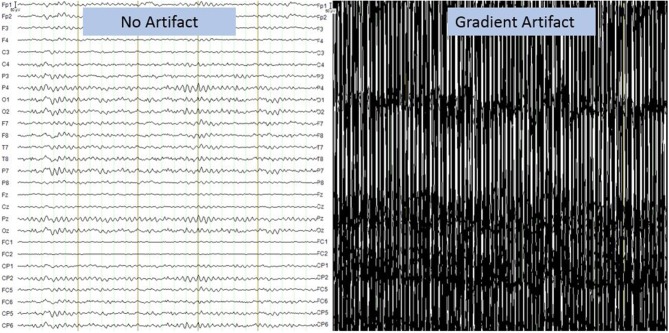
Gradient artifact on electroencephalographic recording. It presents a broadband artifact covering the entire spectrum of EEG frequencies. The amplitude of the artifact is more than 1,000 times that of the EEG signal. Image obtained on a 40 years-old healthy volunteer with hybrid EEG-fMRI system and included for illustrative purpose only.

**Figure 3:**
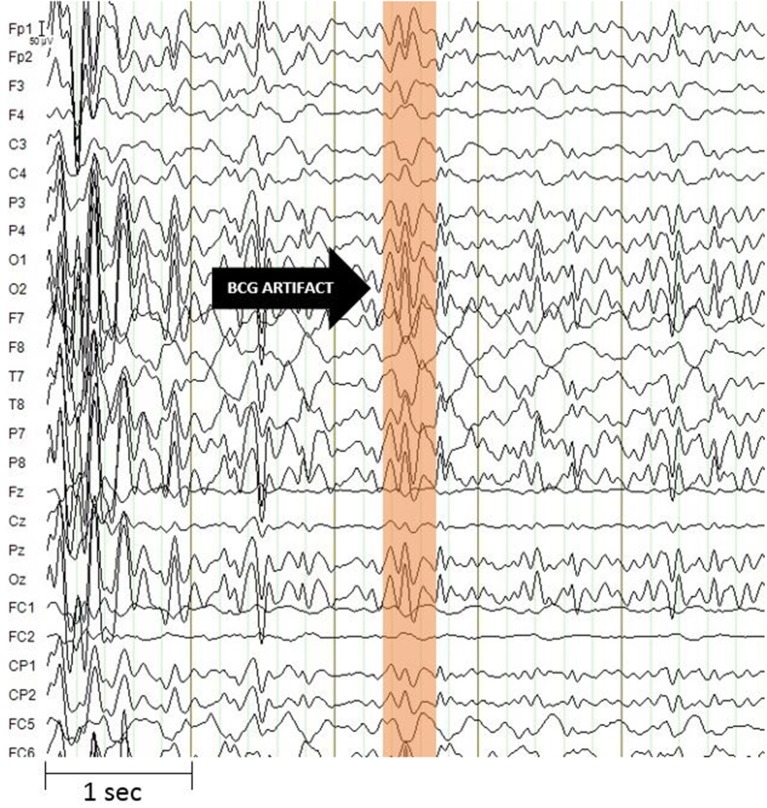
Ballistocardiogram artifact. It has a maximum amplitude of about 100 microvolt and is most evident in the frequency range up to 30 Hz. The artifact undergoes spatio-temporal variability linked to cardiac activity. Image obtained on a 40 years-old healthy volunteer with hybrid EEG-fMRI system and included for illustrative purpose only.

Moreover, the data obtained from the simultaneous acquisition of EEG-fMRI are strongly influenced by artifacts. On the one hand the presence of the helmet generates a variation in the homogeneity of the magnetic field that involved a variation in images quality, on the other hand the presence of the magnetic field itself generates broad-band artifacts, which almost completely cover the electroencephalographic signal (Figure 2) (41). Moreover, the movement of the electrodes caused by pulse-related in the static magnetic field generates a ballistocardiogram artifact that is influenced by the spatio-temporal variability of cardiac cycles in place during recording (Figure 3) (42). For this reason, researchers developed different methods to remove artifact, such as independent component analysis (ICA), that is considered the best method for remove ballistocardiogram artifact (43), or Fourier transform that can be used to correct gradient artifacts (44).

**Figure 4:**
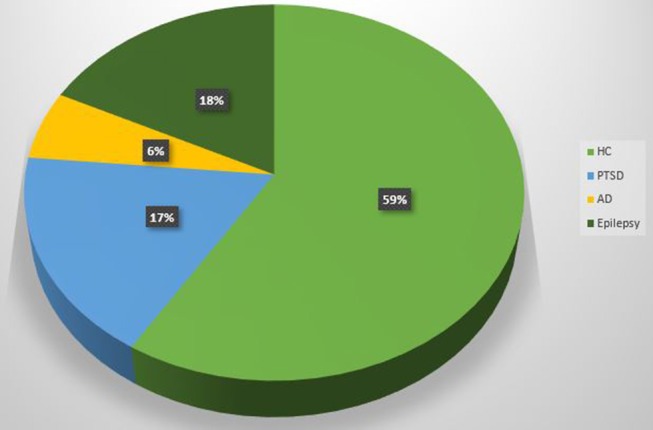
Pie chart. Proportion of EEG-fMRI studies in relation to neuropsychological impairments and healthy control subjects.

Scientific articles published since 2014 on PubMed website, using as key word “simultaneous EEG-fMRI” have been collected in order to include studies with simultaneous acquisitions of EEG-fMRI both in resting state and during tasks execution (Figure 4).

### Simultaneous Resting-State EEG-fMRI

Brain is a dynamic system that generates activity even in a state of rest (Table 1). This can be revealed by EEG recording through the detection of neural waves with different frequency and amplitude and by fMRI through the estimation of different resting state networks linked to specific cerebral functions. The simultaneous acquisition of rsfMRI and EEG makes it possible to consider the brain as a series of systems or networks that interact with each other (47, 51). The interactions are dependent by the concurrent variation of BOLD fluctuations and brain electrical activities. There are many fields of application of simultaneous acquisition of rsfMRI and EEG (Table 1). First studies have focused on methodological issues in healthy subjects, analyzing the reconstruction of EEG signal sources, based on fMRI information, and mainly oriented to a connectivity analysis. However, it is considered necessary to implement the study sample in order to validate the theory (47). The authors demonstrated that simultaneous approach using a 64 channel MR-compatible EEG cap in seventeen adult volunteers is useful to validate whole-brain connectomes extracted by each modality and to elaborate predictive model of dynamic functional connectivity (47). Another study (36) correlated theta and delta frequencies of the temporal lobe with simultaneous fMRI acquisition in fourteen healthy sleep-deprived subjects in awake and drowsy states. The study identified, for the first time, a different brain regional source for the delta and theta rhythms, although their analysis also includes the fastest rhythms, such as alpha, beta and gamma. This kind of approach produces a greater differentiation of the slow rhythms, but decreases the localization of the sources generating different EEG bands. The electrical-BOLD correlation seemed to be stronger for frequencies lower than 1 Hz, and influenced by the spatial relationship between the resting state networks analyzed and the recording zones (48). This relationship has also been used to investigate the basis of some specific electrical oscillations such as the mu rhythm. In a study conducted on thirty-six healthy subjects, simultaneous acquisition of EEG-fMRI has allowed to identify a positive correlation between the power of mu rhythm and the BOLD signal in areas including the anterior cingulate cortex and the anterior insula, confirming the multiple origin of this specific rhythm (50). Concerning neurological diseases applications, a study on eighteen subjects affected by juvenile myoclonic epilepsy demonstrated the added value of the EEG-fMRI acquisition to unveil the pathophysiology of the disease, highlighting the relationships between the frontal networks and the epileptic discharges (46). Another study (45) detected a reduced association between occipital alpha band power and the fluctuation of the BOLD signal in frontal and temporal cortices and in the thalami of fourteen AD patients. In psychiatry, other authors demonstrated a close relationship between the temporal dynamics of default mode network and post-traumatic stress disorder (PTSD) severity in thirty-six veterans, compared to twenty combat-exposed controls (49). It becomes clear that the simultaneous recording of EEG-fMRI can give substantial information on the relationships between the hemodynamic response and neuronal activity. In particular, the resting state acquisition can be fundamental for underling the variability of brain activity and above all to define the structures generally involved in the triggering EEG waves in resting state. In this case, increasing the sample size and using different methods of analysis could validate previous results and disentangle inconsistent or controversial findings.

**Table 1:** A summary of the resting state EEG-fMRI studies since 2014.

**References**	**Application field**	**Subjects**	**MR field**	**MR sequence details**	**EEG**	**preprocessing**	**Results**
Bruggen et al. (45)	AD	14 AD patients and Healthy control	3T	EPI TR/TE:2.5 s/30 ms RES: 3.5 × 3.5 × 3.5 mm	Brain Products 32- channels	EEG: Brain Vision Analyzer for BCG and GA Filter 0.5–70 Hz Notch 50 Hz; MRI: Spm (Matworks and VBM8 toolbox)	Diminished positive association between alpha band power fluctuation and BOLD signal fluctuation in several brain region of AD patient compared to healthy controls
Dong et al. (46)	JME	18 Jouvenil Myoclonical Epilepsy	3T	TR/TE: 52,000 ms/30 ms RES: 1 × 1 × 1	Neuroscan 62- channels	EEG: Curry 7 (Neuroscan software) MRI: SPM8	Evidence of complex discharge-affecting networks in JME patients, in which linear and nonlinear relationships between EEG and fMRI features existed.
Deligianni et al. (47)	Connectivity	17 Healthy Volunteers	1.5T	EPI sequence TR/TE = 2,160/30 ms, 3.3 × 3.3 × 4.0 mm	Electrode cap (BrainCap MR,) 64 channel	EEG: Brain Vision Analyzer 2, SPM12b MRI: Freesurfer, SPM12b	Correlation between the EEG signals and the anatomical zones from which they are generated.
Marawar et al. (36)	Sleep	14 Healthy sleep-deprived subjects	3T	EPI sequence TR/TE = 2000 /30 ms RES:4 × 4 × 4 mm	fEEG; Kappametrics Inc, Chantilly, VA	EEG: MATLAB, MRI: FEAT, FSL	Different correlations for the Delta and Theta rhythms
Keinanen et al. (48)	Epilepsy	10 Healthy controls; 10 patients with drug-resistant epilepsy (DRE).	3T	MREG TE/TE: 100/35 ms RES: 4.5 mm	BrainAmp system with 32 Ag/AgCl electrodes	EEG: Brain Vision Analyzer (version 2.0, Brain Products); MRI: FSL pipeline	Intrinsic brain pulsations play a role in DRE and critically sampled fMRI may provide a powerful tool for their identification.
Yuan et al. (49)	PTSD	36 PTSD; 20 combat-exposed(controls)	3T	EPI sequence (TR/TE) = 2,000/30 ms RES: 1.875 × 1.875 × 2.9 mm	BrainAmp MR Plus amplifiers (Brain Products) 32ch	EEG: BrainVision Analyzer software; MRI: AFNI, RETROICOR,Advanced Normalization Tools	Correspondence between the temporal dynamics of default mode network and PTSD severity
Yin et al. (50)	motor control	36 Healthy Volunteers	3T	(EPI) TR/TE = 1,980/30 ms RES: 3.50 mm	32-channel MR-compatible EEG system (Brain Products)	EEG: Brain Vision Analyzer 2.0 RMI: SMP 5	Power of Mu rhythms positively correlated with BOLD within the anterior cingulate cortex and the anterior insula.

### Simultaneous Task EEG-fMRI

The execution of tasks allows to establish, according to the cognitive domain studied, which cerebral areas are assigned to the specific task (Table 2). According to studies performed with a recognition memory task, EEG-fMRI experiments have demonstrated that theta-alpha low frequency oscillations (4–13 Hz) are linked to the functional activation of a network involving the hippocampus, the striatum and the pre-frontal cortex. These findings confirmed the theory that the hippocampus acts as a modulator of brain activity by acting through low frequency oscillations (52). Hippocampus seems to have an important role also during sleep. In fact, it was demonstrated that hippocampus activity increases during light sleep in relationship with alpha activity (58). It could confirm the idea that memory fixation could occur in light sleep phases, although the acquired subjects had not performed any learning task (58). As for decision making assessment, a simultaneous approach has been employed to investigate common neural substrates for perceptual decisions and accumulation of evidences, highlighting a common role for the posterior medial frontal cortex in both the processes (54). In another study using a two-choice decision-making paradigm, the authors demonstrate that an increase in theta band power, associated with a choice with a negative feedback, corresponds to the activation of fronto-parietal areas; at contrary, an increase in the power of the beta band, associated with a positive feedback, reflects the activation of subcortical are as involved in the reward network (55). Other authors employed the gambling task paradigm in 20 healthy controls to analyze the concurrent activation of large areas related to the reward and punishment, such as posterior cingulate, medial pre-frontal cortex and ventral striatum (56).

**Table 2:** A summary of the task EEG-fMRI studies since 2014.

**Reference**	**Application Field**	**Subjects**	**MR field**	**MR sequence details**	**EEG**	**preprocessing**	**TASK**	**Results**
Herweg et al. (52)	Behavioral/ Cognitive	19 Healthy Control	3T	EPI TR/TE = 4,000/25 ms RES: 2 × 2 × 2 mm	Braincap MR; Brain Products 64-channels	MRI: SPM12b EEG: BrainVision Analyzer 2.0; EEGLAB	Recognition memory task inside of the scanner.	Theta-alpha power is linked to hippocampal connectivity with the striatum and PFC
Zotev et al. (53)	Neurofeedback	15 Healthy Control	3T	EPI TR/TE = 5.0/1.9 ms RES: 0.94 × 0.94 × 1.2 mm 3 mm	Brain Products 32-channels	EEG: BrainVision Analyzer 2.1 software Frmi: AFNI	Retrival of happy autobiographical monets	Emotional control training can improve alpha activity and functional connectivity of amygdala and prefrontal cortex
Pisauro et al. (54)	Neuroscience	21 Healthy Control	3T	EPI TR/TE = 2.5 s/40 ms RES: 3 × 3 mm	Brain Amps MR-Plus 64-channels	EEG: Matlab MRI: FMRIB's Software Library	Independent reward-based decision-making task	task-dependent correlation with the ventromedial prefrontal cortex and the striatum
Andreou et al. (55)	Translational Psychiatry	22 Healthy Control	3T	EPI TR/TE = 2,000/ TE = 25 ms RES: 1 × 1 × 1 mm	BrainVision Recorder 64channel	EEG: Brain Vision Analyzer Version 2.0 MRI: SPM 12	Gambling Task	Negative feedback: Increase in theta band power associated that correspond with activation of fronto-parietal areas. Positive feedback: Increasing in beta band power that reflect activation of subcortical areas
Guo et al. (56)	Neuropsychology	20 Healthy Control	3T	EPI TR/TE = 2,000 ms/35 ms RES: 1 × 1 × 1 mm	Net Station (EEG Electrical Geodesics) 64-channels	EEG: Net Station Software MRI: SPM8	Monerary gambling task	Egg-fMRI acquisition during gambling task underline activation of a posterior cingulate, medial pre-frontal cortex and ventral striatum
Zotev et al. (53)	Neurofeedback	30 Patients with PTSD	3T	EPI TR/TE = 2,000/30 ms RES: 1.875 × 1.875 × 2.9 mm	Brain Products 32 Channels	EEG: BrainVision Analyzer 2.1 software Frmi: AFNI	Think of and write down five happy autobiographical memories.	rtfMRI-nf of the amygdala activity has the potential to correct the amygdala-prefrontal functional connectivity deficiencies specific to PTSD
Zich et al. (57)	Neuropsychology	24 Healthy Control	3T	EPI TR/TE = 1.5 s/2.52 ms RES: 3.1 × 3.1 × 3.0 mm	Brain Product 32-channels	EEG: Brain Vision Analyzer RMI: spm8	Eeg neurofeedback- motor task	Indicate a complex relationship between MI EEG signals and sensorimotor cortical activity and support the role of MI EEG feedback in motor rehabilitation.

A further application of EEG-fMRI is represented by neuro feedback, which allows the modulation of the brain activities, although up to now the information that come back to the patient belong to only EEG (57) or fMRI scan (53, 56, 59). As for EEG neurofeedback, several authors have compared brain activation during motion imaginations and movement execution in healthy subjects, suggesting a role for this approach in the rehabilitation of patients affected by post-stroke paralysis (57). As for fMRI neurofeedback, two studies have investigated the correlation between EEG rhythms and BOLD signal following behavioral modulation. The first one, in a sample of 34 healthy subjects, reported that the modulation of thalamic nuclei activation during the retrieval of happy autobiographical memories, is able to modulate both the alpha activity and the BOLD signal (59). The second one, performed by the same group, in a population of patients affected byPTSD, showed that emotional control training can improve the alpha rhythm and the functional connectivity between the amygdala and the prefrontal cortex, and this enhancement was correlated with a better clinical performance (53).

Up to now, only one article reported the implementation of a novel simultaneous real time fMRI and EEG neurofeedback (60). The authors demonstrated that the training of emotional self-regulation in healthy subjects, based on retrieval of happy autobiographical memories, can modulate both amygdala BOLD fMRI activation and beta band EEG power asymmetry (60). Summarizing, major evidences derived from task-related EEG-fMRI focus on emotional and cognitive processes. This certainly represents a great starting point for understanding and discovering everything concerning psychiatric and neurological syndromes that still remain a big question mark. Although the multimodal approach determines several issues that can complicate the research process, simultaneous EEG-fMRI acquisition remains one of the most appreciated approach, which certainly allows a complete view of brain activity, without affecting the state of patients and subjects participating in the study.

## EEG-fMRI Analysis Methods

**Figure 5:**
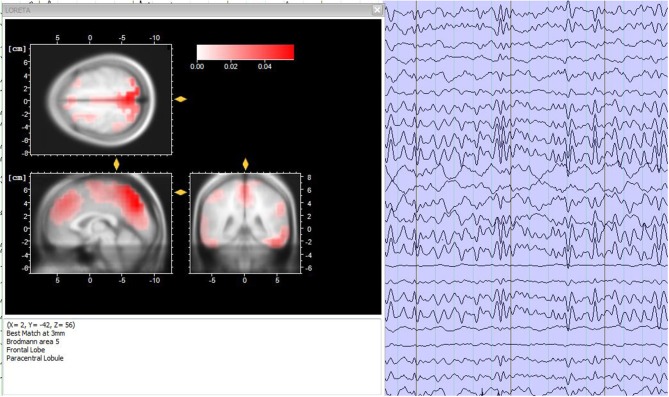
Source analysis. **(Left)** Sources localization of the EEG frequencies for a time period of 3 s, accomplished through the LORETA analysis. **(Right)** 3-s period electroencephalographic pattern of a healthy subject. Image obtained on a 40 years-old healthy volunteer with hybrid EEG-fMRI system and included for illustrative purpose only.

Data analysis is a fundamental step for EEG-fMRI research studies and, in general, for simultaneous multimodal acquisitions. The various analysis used can be contained in two macro-areas: symmetric analysis and integrated analysis (61). Briefly, the symmetrical approach involves the simultaneous analysis of the data extracted from the two methods, while the integrated analysis exploits the data collected by one of the two methods, to understand and validate the data collected from the other one. In this way, it is possible to generate a unique model that facilitates the understanding of brain activity (62). In particular, integrated analyzes include two methods of applications: EEG-informed fMRI (63) and fMRI-informed EEG (64). The first one uses brain electrical activity to predict hemodynamic variations (15). The second one, uses the activation maps extracted by the fMRI to correct and analysis the EEG sources (Figure 5) (65).

Nevertheless, the optimal procedure for the analysis of the simultaneous EEG-fMRI data is still an open issue that needs further investigation in order to extract meaningful quantitative biomarkers, useful to characterize physiological and pathological brain activity, taking advantages by mutual information.

## Future Perspectives

Even if MRI and EEG complement each other considering their different spatial and temporal resolution, the characterization of molecular processes that subtend resting state analysis or a specific task is not achievable through these tools. For this reason, a trimodal approach integrating an MR-compatible EEG-system in the hybrid MR–PET scanner has been proposed and successfully implemented (66). The trimodal acquisition certainly allows a broader and integrative view of the brain activity, although technical issues derived by the PET attenuation of the EEG cap are debated (64, 65).

In an exploratory pilot study, 10 healthy subjects are analyzed in order to implement the value of the single technique and explore the human brain through the different information provided with the same physiological and psychological condition of the subject. The results of these early studies pave the way for further research on different patient populations to exploit the mutual clinical potential of the methods (69).

This kind of approach appeared promising, ensuring the same physiological conditions for all measurements, with the possibility to acquire other synergistic information like perfusion and diffusion changes via MR-based methods.

## Conclusions

Simultaneous EEG-fMRI acquisition represents a reference tool to evaluate the correlation between brain electrical activity and BOLD signal. This technique appeared essential to investigate physiological brain networks in healthy subjects, introducing new evidences about the electrical neural activity and the neurovascular coupling underpinning the BOLD signal. Moreover, it offers the possibility to characterize the relationship between EEG spectrum and regional brain activation, providing new insights on neurological and psychiatric diseases and, hopefully, new treatment targets.

Despite the increasing use of EEG-fMRI, as other multimodal techniques, the question about the optimal integrated and standardized analysis is still open, representing the true challenge that follows the technological development.

## Author Contributions

GM and CC substantial contributed to the conception and design of the work. GM, VA, and MO prepared the literature database and drafted the work. CC, MS, and MA revised critically the manuscript for important intellectual content. MS provided approval for publication of the content.

### Conflict of Interest Statement

The authors declare that the research was conducted in the absence of any commercial or financial relationships that could be construed as a potential conflict of interest.

## Abbreviations

CT: Computed Tomography
MRI: Magnetic Resonance Imaging
PET: Positron Emission Tomography
EEG: Electroencephalography
fMRI: Functional magnetic resonance imaging
BOLD: Blood oxygenation-level-dependent
rsfMRI: resting state functional Magnetic Resonance Imaging
JME: Juvenile Myoclonic Epilepsy
DMN: Default Mode Network
BNG: Basal Ganglia
SRN: Self-reference
PTSD: Post-Traumatic Stress Disorder
AD: Alzheimer Disease
SM: Multiple Sclerosis.
